# Comparison of tumor mutation burden of 300 various non-Hodgkin lymphomas using panel based massively parallel sequencing

**DOI:** 10.1186/s12885-021-08695-7

**Published:** 2021-08-30

**Authors:** Junhun Cho, Sang Eun Yoon, Seok Jin Kim, Young Hyeh Ko, Won Seog Kim

**Affiliations:** 1 Department of Pathology, Samsung Medical Center, Sungkyunkwan University School of Medicine, Seoul, South Korea; 2 Division of Hematology and Oncology, Department of Medicine, Samsung Medical Center, Sungkyunkwan University School of Medicine, #81, Irwon-ro, Gangnam-Gu, Seoul, 06351 South Korea; 3 Department of Pathology, Korea University Guro hospital, #148, Gurodong-ro, Guro-gu, Seoul, 08308 South Korea; 4 Department of Pathology, Hanyang University hospital, #222-1, Wangsimni-ro, Seongdong-gu, Seoul, 04763 South Korea

**Keywords:** Lymphoma, Tumor mutation burden, Massively parallel sequencing, Immunotherapy, Biomarker

## Abstract

**Background:**

Tumor mutation burden is an emerging biomarker for immunotherapy. Although several clinical trials for immunotherapy in lymphoma have been carried out, the mutation burden of various lymphomas is not well known yet. Thus, the objective of this study was to compare tumor mutation burden of various non-Hodgkin lymphomas using panel based massively parallel sequencing.

**Methods:**

We conducted 405 gene panel based massively parallel sequencing of 300 non-Hodgkin lymphomas and investigate the number of SNV/Indel in each lymphoma.

**Results:**

The number of SNV/Indel was higher in mature B-cell lymphoma than in mature T- and NK-cell lymphoma. (*P* < 0.001) The number of SNV/Indel in primary mediastinal large B-cell lymphoma and primary diffuse large B-cell lymphoma of the central nervous system was the highest, which was significantly higher than that in diffuse large B-cell lymphoma, not otherwise specified (DLBCL NOS).(*P* = 0.030 and *P* = 0.008, respectively) The SNV/Indel number in EBV-positive DLBCL NOS was significantly lower than that in DLBCL NOS. (*P* = 0.048) Peripheral T-cell lymphoma, NOS showed no significant difference in the number of SNV/Indel from extranodal NK/T-cell lymphoma, nasal type (*P* = 0.942) or angioimmunoblastic T-cell lymphoma (*P* = 0.739). The number of SNV/Indel in anaplastic large cell lymphoma, ALK-positive was significantly lower than that in anaplastic large cell lymphoma, ALK-negative (*P* = 0.049). It was the lowest among all the lymphomas considered.

**Conclusion:**

Various lymphomas have different mutation burdens. Thus, tumor mutation burden can be used as a promising biomarker for immunotherapy in lymphomas.

**Supplementary Information:**

The online version contains supplementary material available at 10.1186/s12885-021-08695-7.

## Background

Tumor mutation burden (TMB) is one of the most valuable biomarkers for identifying patients who are likely to respond to immune checkpoint blockade [1–4]. Tumor cells harboring more mutations have a higher chance of producing neoantigens that are recognized and targeted by the host immune system [5–9]. Host immune cells can be soldiers that kill cancer cells and immune checkpoint blockades can upregulate the anti-tumor activity of host immune cells, such as cytotoxic T-cells [10–12].

TMB is not only a biomarker to predict the response to immunotherapy, but also has several other meanings. The number of mutations varies across tumor types. It can reflect a different mutational signature and tumorigenesis of each malignancy [1, 13–16]. For example, cutaneous squamous cell carcinomas have higher TMB, whereas uterine cervix squamous cell carcinomas have lower TMB [13]. This difference is due to the fact that etiologies of these two squamous cell carcinomas are different, i.e., ultraviolet light for skin cancer and human papillomavirus infection for uterine cervix cancer. Moreover, the number of mutations can reflect the progression of neoplasms. In general, if the neoplasm progresses, mutations are likely to accumulate. This is not only due to the instability of DNA, but also due to tumor evolution for evading immune surveillance and for cancer survival [17–19]. In this respect, comparing the number of mutations of different tumors and within the same tumor group can provide interesting information about these malignancies.

Immunotherapy has emerged as an important treatment modality not only in carcinomas and melanomas, but also in lymphoid malignancies [20, 21]. To date, studies on immunotherapy for lymphoid malignancy have been mainly been conducted for classic Hodgkin lymphoma [22–24]. In addition, the effects of immunotherapy for a subset of non-Hodgkin lymphomas have been reported [25, 26]. Although the significance of TMB in lymphomas is increasing, studies on TMB in lymphomas are insufficient. Therefore, the objective of this study was to investigate the number of single nucleotide variant (SNV) and Indel in 300 various non-Hodgkin lymphomas using massively parallel sequencing with a 405-gene panel.

## Methods

### Patient selection

Patients diagnosed with lymphoma at Samsung medical center (Seoul, Korea) were enrolled in the “SMC lymphoma registry” with informed consent. From January 2019, next generation sequencing (NGS) was performed for patients with sufficient tumor sample volumes encompassing those diagnosed for the first time and relapsed/refractory patients. The results for 300 non-Hodgkin lymphoma patients who underwent NGS by December 2020 were analyzed. Pathologic diagnosis was made according to the 2016 WHO classification [27] by two pathologists (JC and YHK).

### Panel based massively parallel sequencing (LymphomaSCAN)

Targeted genetic sequencing was performed using the LymphomaSCAN panel, including 405 genes (Supplementary Table 1). The Samsung medical center operates an NGS platform called XSCAN for various malignancies (CancerSCAN, BrainTumorSCAN, and PedSCAN). The gene panel of LymphomaSCAN was constructed by adding genes known to be associated with hematologic malignancies to a common gene panel shared with other XSCANs for discussions between oncologists and hematopathologists. Extracted genomic DNA was sheared using a Covaris S220 (Covaris, Woburn, MA, USA). Targeted genes were captured using a SureSelect XT Reagent Kit, HSQ (Agilent Technologies) and a paired-end sequencing library was constructed using a barcode. DNA sequencing was performed on a NextSeq 550 Dx sequencer (Illumina, San Diego, CA, USA). The paired-end reads were aligned to the human reference genome (hg19) using BWA-MEM v0.7.5, Samtools v0.1.18, GATK v3.1–1, and Picard v1.93. SNVs were called using MuTect version 1.1.4, LoFreq version 0.6.1, and VarDict version 1.06 software with a variant allele frequency ≥ 1% or a number of variant supporting reads > 4. We manually reviewed variants with supporting reads < 20 using an Integrative Genomics Viewer browser and filtered out sequencing errors. We identified small Indels using Pindel version 0.2.5a4 with a number of variant supporting reads > 9. We further filtered out variants with a minor allele frequency ≥ 1% in the 1000 Genomes Project database [28], the Exome Aggregation Consortium database [29], the National Heart, Lung, and Blood Institute’s Exome Sequencing Project database [30], the Korean Reference Genome Database [31], the Korean Variant Archive [32], and an in-house database of 192 Korean individuals. To measure the number of mutations consistently, only SNV/Indel results were used, whereas copy number variation and fusion results were discarded. To filter out false-positive results, variants with a variant allele frequency (VAF) < 5% and total reads < 100 were excluded.

### Statistical analysis

We used the SPSS 27 statistical software program (IBM Corporation) for all statistical analyses. The Mann–Whitney U test was performed to test the difference between the TMB for two tumors. Two-tailed *P* values < 0.05 were considered statistically significant.

## Results

### Patient characteristics

The median age of the 300 patients was 58 years (range, 19–90 years). There were 187 males and 113 females (male to female ratio: 1.65:1) (Table 1). Among the 300 lymphomas, there were 243 (81.0%) mature B-cell neoplasms, 53 (17.6%) mature T- and NK-cell neoplasms, and 4 (1.3%) precursor lymphoid neoplasms. Among mature B-cell neoplasms, diffuse large B-cell lymphoma, not otherwise specified (DLBCL NOS) was the most common, with 154 patients, followed by follicular lymphoma in 29 patients, primary diffuse large B-cell lymphoma of the central nervous system (CNS DLBCL) in 17 patients, and mantle cell lymphoma in 11 patients. Among mature T- and NK-cell neoplasms, extranodal NK/T-cell lymphoma, nasal type (ENKTL) was the most common (18 patients), followed by peripheral T-cell lymphoma, not otherwise specified (PTCL NOS) and angioimmunoblastic T-cell lymphoma (AITL) (10 patients each).

**Table 1:** Clinical characteristics and the number of SNV/Indel by pathologic diagnosis

Diagnosis	Number of patients	Age	M:F	Ann-Arbor stage		Therapeutic status		Number of SNV/Indel		
		median (range)		I or II	III or IV	Pre-Tx. (at diagnosis)	Post-Tx. (relapsed/refractory)	Mean	Median	Range
**Mature B-cell neoplasms**	**243**									
Diffuse large B-cell lymphoma, NOS	154	61 (26–86)	93:61	71	83	125	29	24.84	23	11–87
Primary diffuse large B-cell lymphoma of the CNS	17	62 (34–86)	10:7	17	0	16	1	31.53	30	17–51
Primary mediastinal large B-cell lymphoma	5	36 (25–62)	3:2	1	4	5	0	31.80	32	26–41
EBV-positive diffuse large B-cell lymphoma, NOS	6	62 (19–90)	4:2	2	4	5	1	17.00	18	5–28
High-grade B-cell lymphoma	6	50.5 (37–63)	5:1	1	5	4	2	20.83	21.5	15–27
Burkitt lymphoma	1	69	0:1	0	1	0	1	22.00	22	
Plasmablastic lymphoma	2	58 (52–64)	2:0	1	1	1	1	19.50	19.5	17–22
Follicular lymphoma	29	50 (28–79)	18:11	5	24	27	2	19.62	18	8–35
Mantle cell lymphoma	11	63 (47–80)	8:3	2	9	7	4	19.09	20	11–27
Nodal marginal zone lymphoma	3	59 (58–63)	1:2	2	1	2	1	23.33	16	12–42
Extranodal marginal zone lymphoma of MALT	4	55.5 (44–68)	2:2	3	1	3	1	19.25	20.5	13–23
Lymphoplasmacytic lymphoma	2	59.5 (53–66)	2:0	0	2	0	2	17.00	17	14–20
Chronic lymphocytic leukemia/Small lymphocytic lymphoma	3	56 (53–61)	1:2	0	3	3	0	15.33	16	10–20
**Mature T- and NK-cell neoplasms**	**53**									
Peripheral T-cell lymphoma, NOS	10	48 (25–71)	7:3	3	7	5	5	17.60	18	11–23
Angioimmunoblastaic T-cell lymphoma	10	66.5 (43–81)	5:5	0	10	7	3	18.70	19.5	10–24
Follicular T-cell lymphoma	2	50 (48–52)	2:0	0	2	1	1	14.00	14	13–15
Nodal peripheral T-cell lymphoma with TFH phenotype	4	68.5 (64–75)	3:1	0	4	3	1	14.25	14.5	13–15
Extranodal NK/T-cell lymphoma, nasal type	18	56 (32–79)	11:7	11	7	12	6	17.44	17	9–25
Anaplastaic large cell lymphoms, ALK-positive	5	35 (20–58)	4:1	1	4	4	1	12.20	14	6–16
Anaplastaic large cell lymphoms, ALK-neative	4	40 (29–56)	3:1	1	3	4	0	22.25	23	15–28
**Precursor lymphoid neoplasms**	**4**									
Lymphoblastic leukemia/lymphoma	4	44.5 (34–70)	3:1	1	3	4	0	19.00	19.5	15–22
**Total**	**300**	**58 (19–90)**	**187:113**	**122**	**178**	**238**	**62**	**22.68**	**21**	**5–87**

*SNV* Single nucleotide variant, *NOS* Not otherwise specified

### Number of SNV/Indel in all cases

**Fig. 1:**
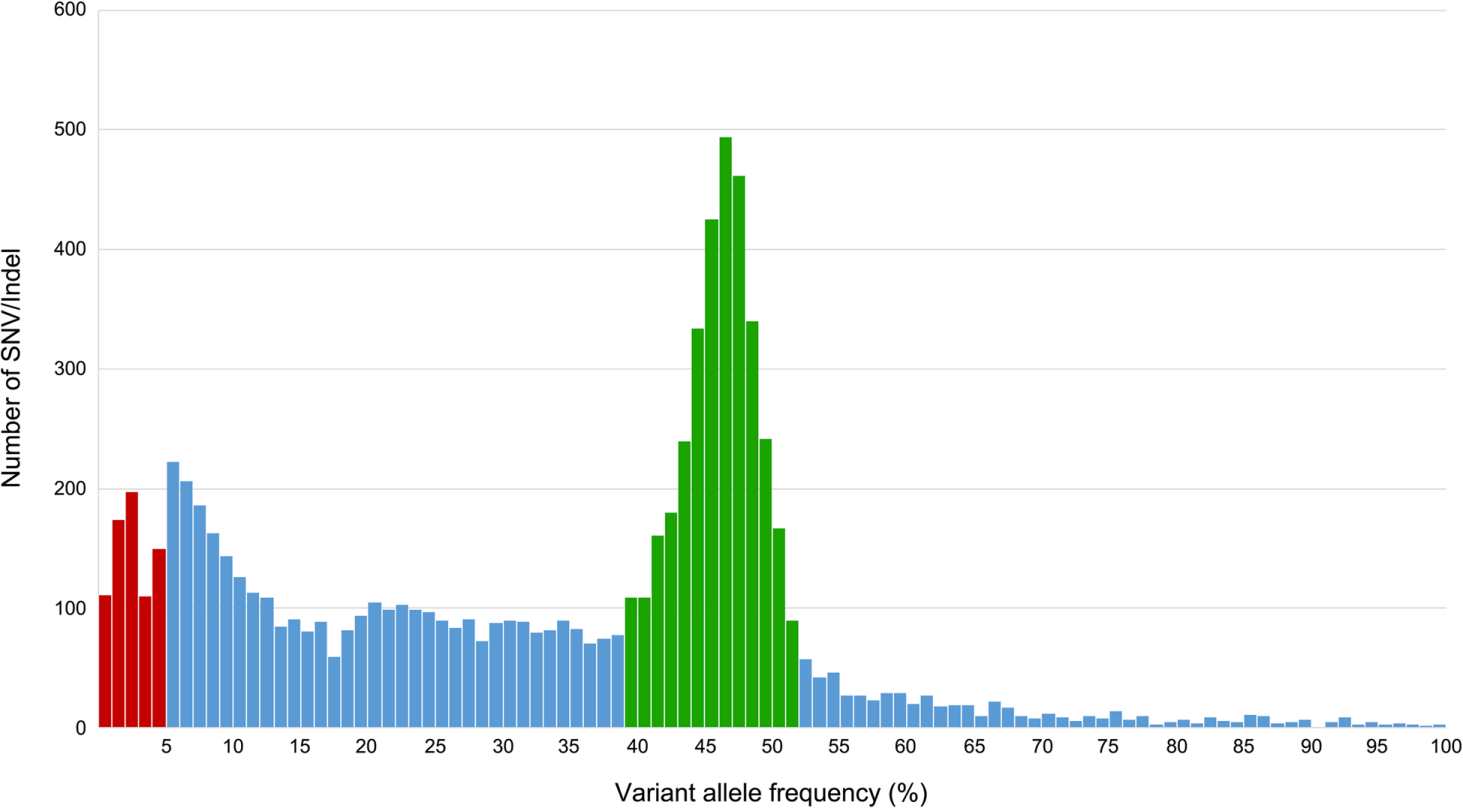
Number of SNV/Indel according to variant allele frequency in 300 cases. Red bars are the section with variant allele frequency less than 5%. Green bars are the section estimated to contain lots of germline mutations

**Fig. 2:**
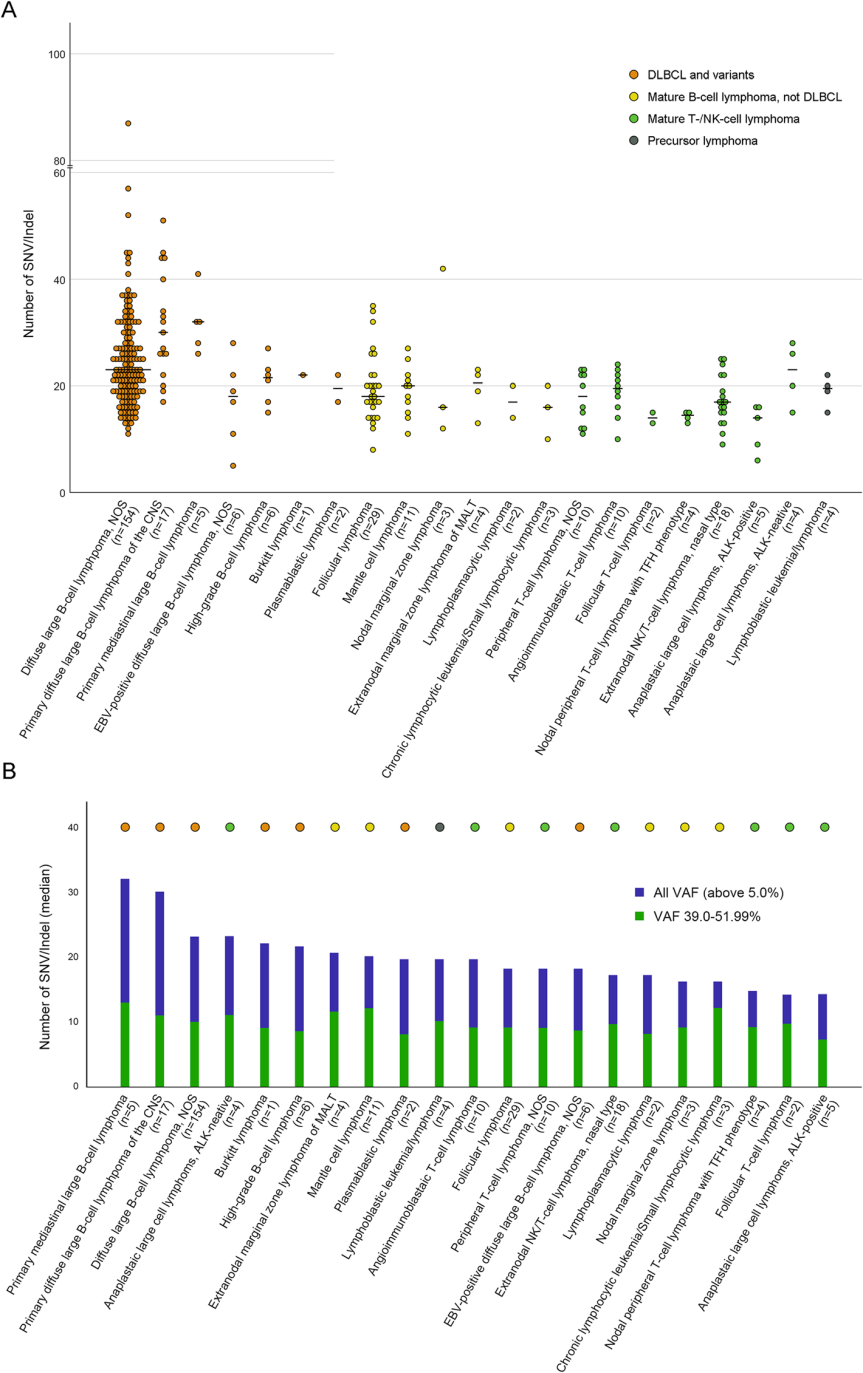
**A** Scatter plot of number of SNV/Indel for all cases. The horizontal bar represents the median value. **B** Bar graph in which the number of SNV/Indel is arranged in the order of median value. Blue bar represents the total number of SNV/Indel and green bar represents the number of SNV/Indel corresponding to the germline-containing VAF interval (39.0–51.99%). The classification of lymphomas is indicated by the color of dots

When the number of SNV/Indel was counted by dividing VAF by 1%, VAF between 39 and 52% was higher than expected (Fig. 1). Therefore, this area was considered a section with a high probability of including germline mutations. Upon excluding VAF < 5% and total read < 100, the average of the number of SNV/Indel was 22.68 (6804/300). The average SNV/Indel number was 23.98 in mature B-cell neoplasms and 17.21 in mature T- and NK-cell neoplasms, with a significant (*P* < 0.001) difference between the two. When all lymphomas were arranged in the order of median value of the number of SNV/Indel mutations, primary mediastinal large B-cell lymphoma (PMLBL) (median: 32) ranked first, followed by CNS DLBCL (median: 30), DLBCL NOS (median: 23), and anaplastic large cell lymphoma, ALK-negative (ALCL, ALK-) (median: 23) (Fig. 2). Lymphomas with the lowest SNV/Indel numbers were in the order of ALCL, ALK-positive (ALCL, ALK+) (median: 14), follicular T-cell lymphoma (median: 14), and nodal peripheral T-cell lymphoma with TFH phenotype (PTCL TFH) (median: 14.5). The number of variants corresponding to the VAF 39–52% section did not show significant difference according to the type of lymphoma (*P* = 0.529) (Fig. 2B).

### Number of SNV/Indel in diffuse large B-cell lymphoma variants

**Fig. 3:**
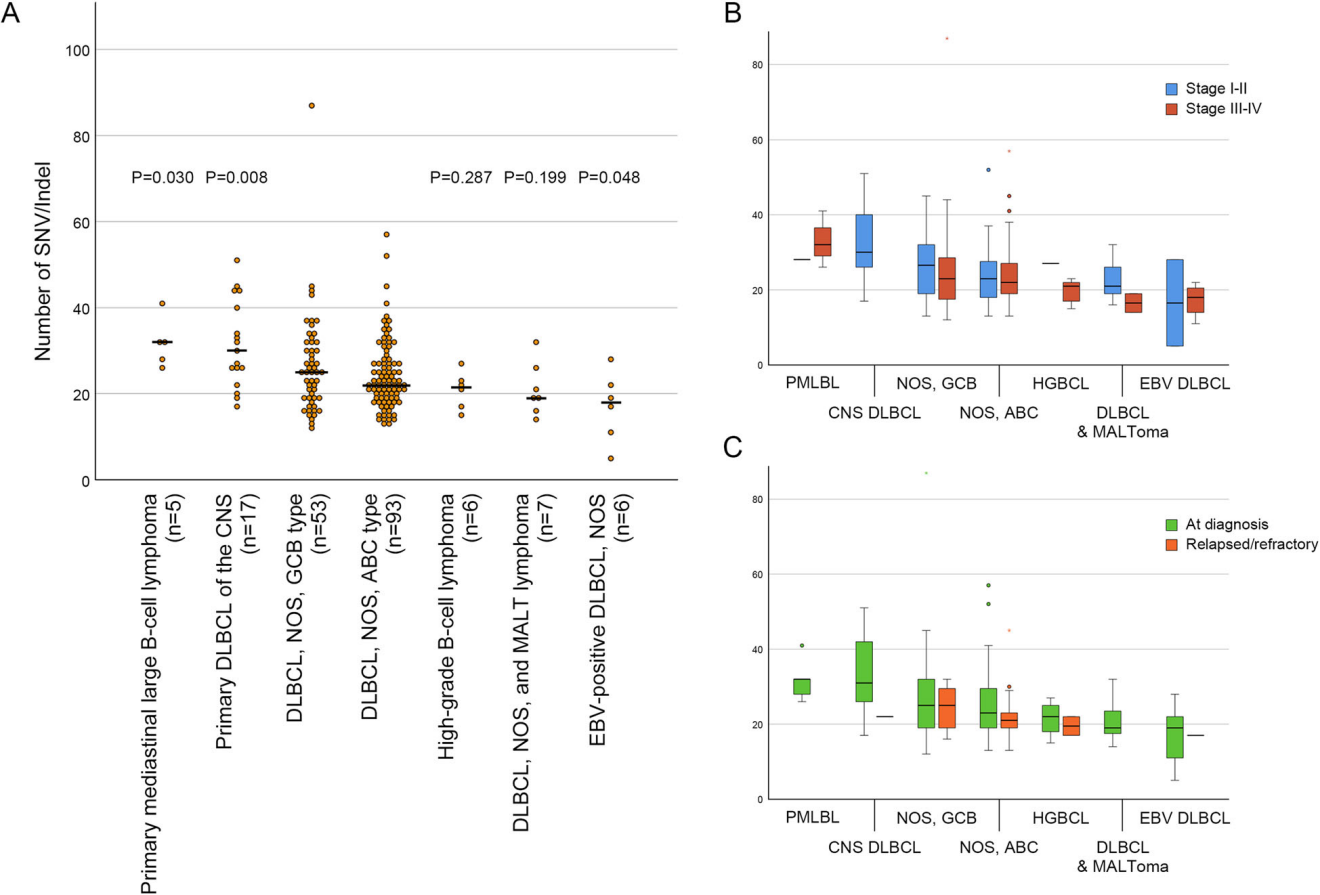
**A** Scatter plot showing SNV/Indel numbers of diffuse large B-cell lymphoma (DLBCL) variants. Horizontal bar represents the median value. The *P* value above each lymphoma is the result of Mann-Whitney U test in comparison with DLBCL, not otherwise specified (germinal center B-cell type + activated B-cell type). **B** Box plot comparing SNV/Indel numbers of DLBCL variants by Ann-Arbor stage. **C** Box plot comparing SNV/Indel numbers of DLBCL variants between tumors at diagnosis and relapsed/refractory tumors. DLBCL, diffuse large B-cell lymphoma; CNS, central nervous system; NOS, not otherwise specified, GCB, germinal center B-cell; ABC, activated B-cell; MALT, mucosa-associated lymphoid tissue, EBV, Epstein-Barr virus; PMLBL, primary mediastinal large B-cell lymphoma, HGBCL, high-grade B-cell lymphoma

Regarding types of DLBCL NOS according to Hans’ classification [33], 93 cases had a post-germinal center B-cell type (activated B-cell type, ABC type) and 53 cases had a germinal center B-cell type (GCB type), with no significant difference in the number of SNV/Indel between these two types of DLBCL NOS. (*P* = 0.308) (Fig. 3A) Compared with DLBCL NOS, PMLBL (*P* = 0.030) and CNS DLBCL (*P* = 0.008) had more SNV/Indel mutations, whereas EBV-positive diffuse large B-cell lymphoma, not otherwise specified (EBV DLBCL) had significantly (*P* = 0.048) less SNV/Indel mutations. Even after performing Bonferroni correction five time to correct for multiple testing (*P* values < 0.01 were considered statistically significant), the number of SNV/Indels in CNS DLBCL was significantly higher than that in DLBCL NOS. High grade B-cell lymphoma (HGBCL) (*P* = 0.287), including double-hit lymphoma and triple-hit lymphoma, and DLBCL NOS admixed with extranodal marginal zone lymphoma of mucosa-associated lymphoid tissue (MALT lymphoma), showed no significant (*P* = 0.199) difference in SNV/Indel number compared with DLBCL NOS. There was no significant difference in SNV/Indel number according to the Ann Arbor stage (Fig. 3B) and therapeutic status (Fig. 3C) of DLBCL variants.

### Number of SNV/Indel in mature B-cell lymphomas other than DLBCL variants

**Fig. 4:**
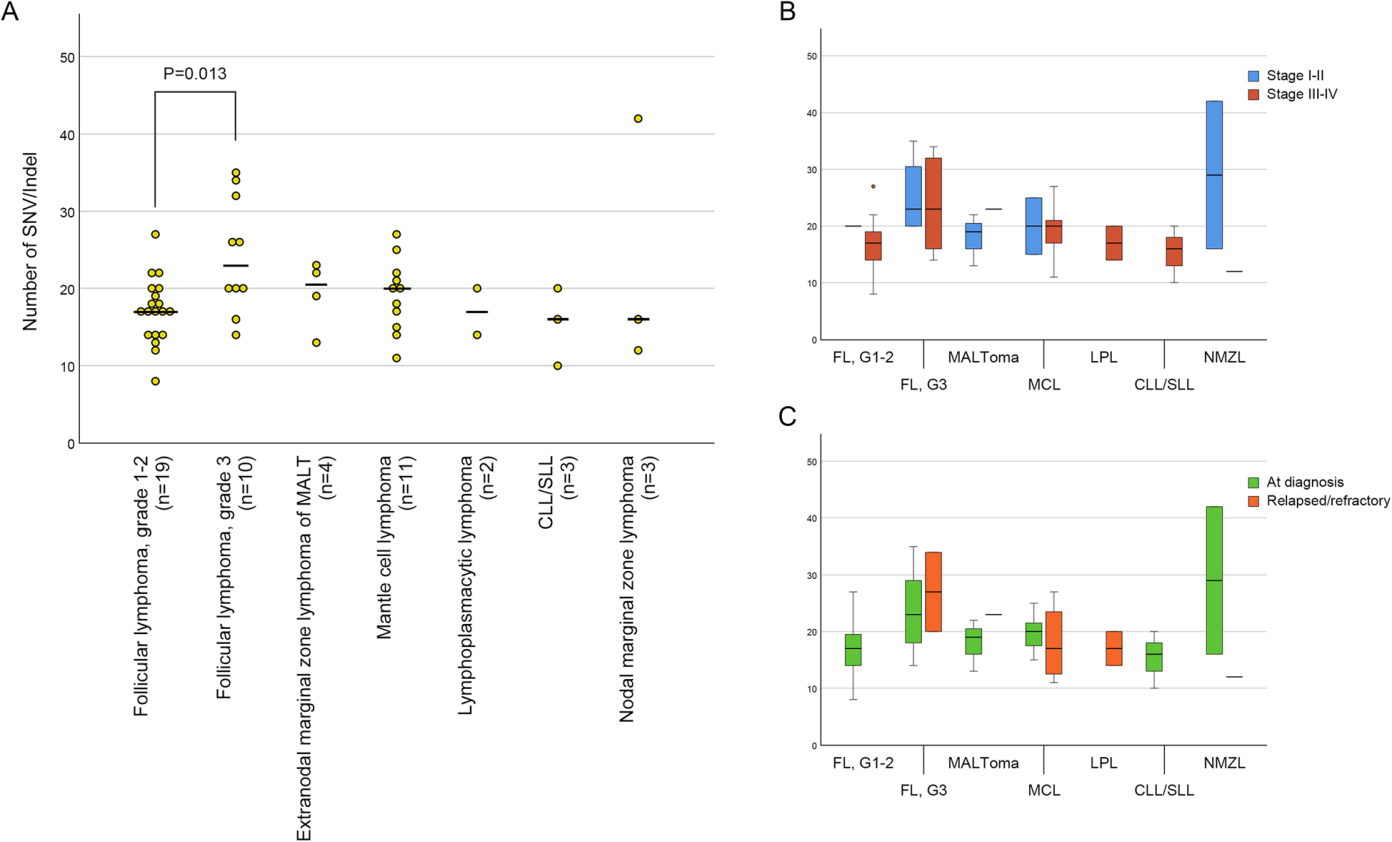
**A** Scatter plot showing SNV/Indel numbers of mature B-cell lymphoma except diffuse large B-cell lymphoma (DLBCL) variants. Horizontal bar represents the median value. **B** Box plot comparing SNV/Indel numbers of mature B-cell lymphoma except DLBCL variants by Ann-Arbor stage. **C** Box plot comparing SNV/Indel numbers of mature B-cell lymphoma except DLBCL variants between tumors at diagnosis and relapsed/refractory tumors. MALT, mucosa-associated lymphoid tissue; CLL/SLL, chronic lymphocytic leukemia/small lymphocytic lymphoma; FL, follicular lymphoma; MALToma, extranodal marginal zone lymphoma of MALT; MCL, mantle cell lymphoma; LPL, lymphoplasmacytic lymphoma; NMZL, nodal marginal zone lymphoma

The results of a comparison of the number of SNV/Indel of mature B-cell lymphomas other than DLBCL variants are depicted in Fig. 4A. When follicular lymphoma was classified as low grade (grade 1–2, *n* = 19) and high grade (grade 3A and 3B, *n* = 10) by histologic grading [27], the number of SNV/indel of high grade follicular lymphoma (median: 20) was significantly (*P* = 0.013) higher than that of low grade follicular lymphoma (median: 17). Mantle cell lymphoma, lymphoplasmacytic lymphoma, chronic lymphocytic leukemia/small lymphocytic lymphoma (CLL/SLL), and nodal marginal zone lymphoma showed no significant differences in the number of SNV/Indel. Compared with DLBCL NOS, low grade follicular lymphoma (*P* < 0.001) and mantle cell lymphoma (*P* = 0.026) had significantly less SNV/Indel numbers whereas high grade follicular lymphoma showed no significant difference (*P* = 0.973). There was no significant difference in SNV/Indel number according to Ann Arbor stage (Fig. 4B) and therapeutic status (Fig. 4C) of mature B-cell lymphomas. The remaining small B-cell lymphomas were not suitable for statistical analysis because of the small number of cases. However, all of them showed lower SNV/Indel median values than mantle cell lymphoma.

### Number of SNV/Indel in mature T- and NK-cell neoplasms

**Fig. 5:**
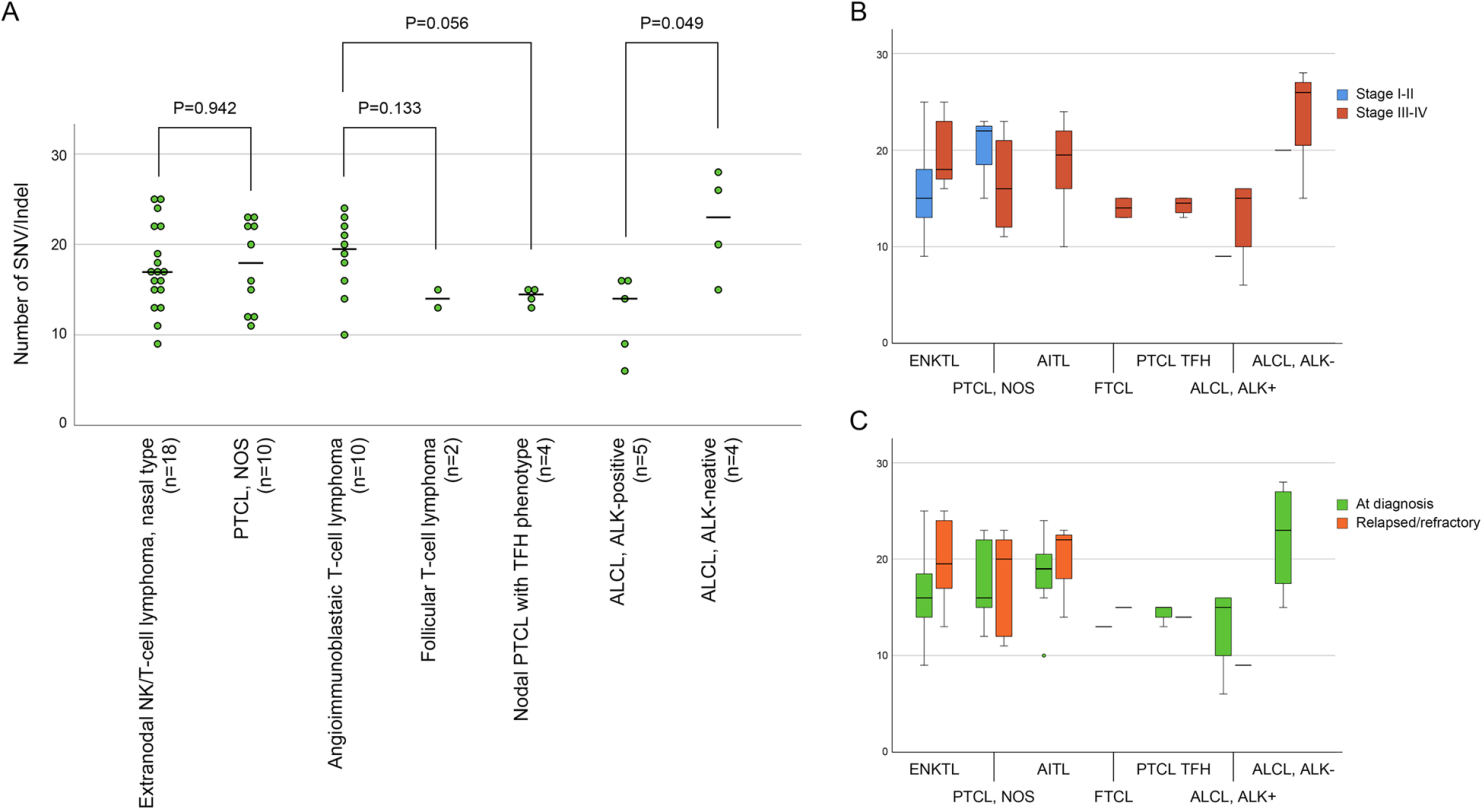
**A** Scatter plot showing SNV/Indel numbers of mature T- and NK- cell lymphomas. Horizontal bar represents the median value. **B** Box plot comparing SNV/Indel numbers of mature T- and NK- cell lymphomas by Ann-Arbor stage. **C** Box plot comparing SNV/Indel numbers of mature T- and NK- cell lymphomas between tumors at diagnosis and relapsed/refractory tumors. PTCL, peripheral T-cell lymphoma; NOS, not otherwise specified; TFH, T follicular helper cell; ALCL, anaplastic large cell lymphoma, ALK, anaplastic lymphoma kinase; ENKTL, extranodal NK/T-cell lymphoma, nasal type; AITL, angioimmunoblastic T-cell lymphoma; FTCL, follicular T-cell lymphoma

PTCL NOS showed no significant difference in SNV/Indel number compared with ENKTL (*P* = 0.942) or AITL (*P* = 0.739) (Fig. 5A). When these three T-cell lymphomas of T follicular helper (TFH) cell origin were compared, the median number of SNV/Indel of AITL was higher than that of follicular T-cell lymphoma (*P* = 0.133) or PTCL TFH (*P* = 0.056), although the difference was not statistically significant. In ALCL, the TMB was significantly higher in ALK-negative than in ALK-positive (*P* = 0.049). PTCL NOS showed a significantly lower SNV/Indel number than DLBCL NOS (*P* = 0.008). There was no significant difference in SNV/Indel number according to Ann Arbor stage (Fig. 5B) and therapeutic status (Fig. 5C) of mature T- and NK-cell lymphomas. ENKTL tended to show more mutations in advanced disease (Ann Arbor stage III or IV) or post-chemotherapy patients (relapsed/refractory), but the difference was not statistically significant.

## Discussion

In this study, we aimed to determine whether there are differences in the TMB of various non-Hodgkin lymphomas across B- and T−/NK-cell lymphomas. Whole exome sequencing (WES) is the gold standard for measuring TMB in cancers. However, because of its high cost, WES is generally performed for research, not for diagnosis. Recently, several studies have shown that a well-designed gene panel can be used to calculate TMB which is very similar to those of WES [13, 34–37]. The gene panel used in this study consists of more than 400 genes designed for lymphomas, however, calculated TMB using them has not been validated through parallel WES. Rather than classifying TMB-high and TMB-low groups of lymphomas using precise cutoff value, we tried to compare the number of genetic mutations of various non-Hodgkin lymphomas using the same platform, and were able to make some meaningful discoveries.

Overall, B-cell lymphomas had more mutations than T-cell lymphomas. This result is consistent with the existing knowledge that B-lymphocytes have a complex maturation process, including somatic hypermutation that T-lymphocytes do not have [38]. Among DLBCL variants, PMLBL and CNS DLBCL had more mutations than DLBCL NOS. Of all the lymphomas included in this study, these two tumors were the only two lymphomas with median SNV/indel number ≥ 30. PMLBL is a distinct subtype of DLBCL variants and is known to have a gene expression profile that overlaps with the profile of classic Hodgkin lymphoma [39, 40]. Some studies have shown that a part of refractory and recurrent PMLBL responds to pembrolizumab, a programmed death-1 (PD-1) blocker [26, 41]. This is thought to be related to the fact that frequent amplification and translocation events occur at 9p24.1, in which *CD274* (PD-L1) gene is located, in PMLBL [42, 43]. In addition, the formation of an immune cell-rich microenvironment, such as classic Hodgkin lymphoma, is also a necessary condition for the action of immune checkpoint blockade. The high TMB observed in PMLBL may also be one of the reasons why this tumor is eligible for immune checkpoint blockade treatment. CNS DLBCL also had a large number of SNV/Indels, comparable to that of PMLBL. According to gene expression profiling analysis of CNS DLBCL, it was not markedly different from systemic DLBCL [44], although it mainly belongs to the post-germinal center B-cell type. Although not included in this study, our NGS results for CNS DLBCL had a higher 9p21 (including *CDKN2A* and *CDKN2B*) loss ratio (13/17, 76.5%) than DLBCL NOS (43/154, 27.9%). In our cohort, CNS DLBCL was considered to be a group with specific features such as high TMB and 9p21 loss, whereas it is genetically included in the spectrum of systemic DLBCL. Owing to the specificity of immune sanctuary site, further studies are needed to determine the effect of high TMB of CNS DLBCL on the effectiveness of immune checkpoint blockades. It has been reported that CNS DLBCL also shows reactivity to pembrolizumab [45]. In contrast, EBV DLBCL showed significantly lower TMB than DLBCL NOS. This supports the hypothesis that EBV infection is a strong driver of tumorigenesis in B-cell lymphoma. In general, TMB-low cancers are considered to be less suitable for immunotherapy. However, apart from the number of mutations, EBV infection can generate neoantigens that can be targets of host immune cells [46]. Therefore, EBV DLBCL patients should not be excluded from candidates for immunotherapy, although they have a low TMB [47].

Mature B-cell lymphomas not included in DLBCL variants had lower TMB than DLBCL NOS, and the difference in SNV/Indel number between them was not significant. The fact that the SNV/Indel number of grade 3 follicular lymphoma was significantly higher than that of grade 1–2 follicular lymphoma suggests that the histologic grade of follicular lymphoma might increase with disease progression. The TMB of high-grade follicular lymphoma was between that of low grade follicular lymphoma and DLBCL NOS. Owing to the ethnic characteristics of our cohort, only three CLL/SLL patients were included. In addition to CLL/SLL, there were only a few samples of small B-cell lymphomas in this study. Therefore, further research using a sufficient number of cases is needed.

Several interesting findings with regard to the TMB in mature T-cell lymphomas were observed. There was no difference in TMB between PTCL NOS and ENKTL. This is in contrast with the significantly lower TMB of EBV DLBCL compared with that of DLBCL NOS in B-cell lymphoma. Although ENKTL is not EBV-positive PTCL per se, this suggests that the role of EBV infection in the tumorigenesis of B-cell lymphoma and T−/NK-cell lymphoma is different [48, 49]. Among those of PTCLs derived from TFH cell, the TMB of AITL tended to be higher than that of the other two (follicular T-cell lymphoma and PTCL TFH), although the difference was not statistically significant. Considering that follicular T-cell lymphoma and PTCL TFH have molecular signatures similar to that of AITL [50], this supports the hypothesis that AITL is a more progressed disease than the other two lymphomas. It is known that ALK-positive and ALK-negative ALCLs share a common molecular signature [51], however, the significantly higher TMB of ALCL ALK- compared with that of ALCL ALK+ suggests that *ALK* gene translocation is a very strong oncogenic event. In addition, it is consistent with the fact that ALCL ALK- generally has poorer clinical outcome than ALCL ALK+, despite their similar histologic morphology [52].

Interestingly, the number of SNV/Indel was not associated with Ann Arbor stage in most lymphomas. In this study, high-grade lymphoma generally had more mutations than low-grade lymphoma, however, there was no difference in the number of mutations between advanced/systemic disease and localized disease in the same diagnosis. These findings suggest that high- or low-grade lymphomas develop via separate pathways but accumulation of mutations is not a major mechanism of disease progression in most lymphomas. Moreover, a history of chemotherapy was not associated with an increase in the number of SNV/Indel. Although the diagnosis of patients in our cohort varied, most patients with relapsed/refractory tumors received CHOP-containing regimen as first-line chemotherapy. Cyclophosphamide and doxorubicin are agents that damage DNA, but they did not actually increase the number of mutations in post-therapeutic lymphoma patients. Further research is needed to understand these observations.

Our study has several limitations. First, we used panel-based target sequencing instead of WES. Although targeted sequencing has been widely used as a method for measuring TMB in various malignancies [34–37], and more than 400 genes were included in our panel, it has not been validated for TMB measurement. For this reason, our study did not suggest a cutoff value for classifying TMB-high and TMB-low group lymphomas. Second, matched normal tissues were not used in the sequencing process. Therefore, germline mutations were not clearly filtered out. Third, copy number variation and translocation results other than SNV/Indel were not included in our analysis. Finally, our study only analyzed the number of SNV/Indel, not specific mutant genes. The number of mutations is determined by a variety of factors, such as different molecular subtypes or mutational signatures even in tumors of same histologic diagnosis. Because these factors may also affect responsiveness to immunotherapy, the pattern of mutations should also be considered in using TMB as a biomarker for immunotherapy. It is expected that through further studies that complement the aforementioned limitations, the TMB of lymphomas can be more accurately analyzed and the TMB-high group lymphomas eligible for immunotherapy can be classified.

## Conclusion

Different types of lymphomas have different numbers of mutations. The number of mutations might reflect the clinical and pathological characteristics of each lymphoma. In general, the number of mutations in B-cell lymphoma is higher than that of T/NK-cell lymphoma, and the number of mutations in high-grade lymphoma is higher than that in low-grade lymphoma. TMB is expected to be a useful biomarker for immunotherapy of various lymphomas, and our findings suggest that patients with high grade B-cell lymphoma (PMLBL, CNS DLBCL etc.) and some T/NK-cell lymphoma (ALCL, ALK-negative etc.) are most likely to respond to immunotherapy. Further clinical research is required to verify whether these results can be applied in patient treatment.

## Supplementary Information

**Additional file 1. **Gene panel of LymphomaSCAN (*n*=405)

## Data Availability

The datasets used and/or analysed during the current study are available from the corresponding author on reasonable request.

## Abbreviations

TMB: Tumor mutation burden
SNV: Single nucleotide variant
NGS: Next generation sequencing
VAF: Variant allele frequency
DLBCL NOS: Diffuse large B-cell lymphoma, not otherwise specified
CNS DLBCL: Primary diffuse large B-cell lymphoma of the central nervous system
ENKTL: Extranodal NK/T-cell lymphoma, nasal type
PTCL NOS: Peripheral T-cell lymphoma, not otherwise specified
AITL: Angioimmunoblastic T-cell lymphoma
PMLBL: Primary mediastinal large B-cell lymphoma
ALCL: Anaplastic large cell lymphoma
PTCL TFH: Nodal peripheral T-cell lymphoma with TFH phenotype
ABC: Activated B-cell
GCB: Germinal center B-cell
EBV DLBCL: EBV-positive diffuse large B-cell lymphoma, not otherwise specified
HGBCL: High grade B-cell lymphoma
MALT lymphoma: Extranodal marginal zone lymphoma of mucosa-associated lymphoid tissue
WES: Whole exome sequencing
