# Supplementary material for: Comparison of tumor mutation burden of 300 various non-Hodgkin lymphomas using panel based massively parallel sequencing
Source: BMC Cancer. 2021 Aug 30;21:972. doi: 10.1186/s12885-021-08695-7 (PMC8404326; doi:10.1186/s12885-021-08695-7)
Supplement: Supplementary file 1 — Additional file 1. Gene panel of LymphomaSCAN (n=405) [file 12885_2021_8695_MOESM1_ESM.docx]

**Supplementary table 1.** Gene panel of LymphomaSCAN (n=405)

===========================================================================================

ABCG2 ABHD17A ABL1 ACTB AHR AKT1 AKT2 AKT3 ALK ANKIB1

ANKRD11 ANO10 APC ARAF ARID1B ARID1A ARID2 ATF7IP ATM ATRX

AURKA AURKB B2M BAP1 BCL10 BCL11A BCL11B BCL2 BCL2L2 BCL6

BCL7A BCOR BCORL1 BCR BIRC3 BLK BRAF BRCA1 BRCA2 BRD2

BRD3 BRD4 BTG1 BTK CACNA1D CARD11 CASP10 CCDC13 CCNA1 CCND1

CCND2 CCND3 CCNE1 CCR4 CCR7 CCT6B CD19 CD22 CD274 CD28

CD37 CD38 CD40 CD58 CD79A CD79B CDH1 CDH9 CDK12 CDK4

CDK6 CDK8 CDKN1B CDKN2A CDKN2B CDKN2C CEP128 CHEK1 CHEK2 CHUK

CIITA CPXM2 CRBN CSF1R CSNK1A1 CTLA4 CTNNB1 CUX1 CXCL10 CXCR4

DAB2IP DAPP1 DDR2 DDX3X DHX16 DIAPH2 DNMT3A DUSP1 DUSP22 DUSP9

EGFR EIF3A EML5 EP300 EPHB1 EPHB4 EPSTI1 ERBB2 ERBB3 ERBB4

ESCO1 ESR1 ETF1 ETS1 ETV1 ETV5 ETV6 EWSR1 EZH2 FAM3C

FANCL FAS FBP1 FBXW7 FGFR1 FGFR2 FGFR3 FGR FLI1 FLNC

FLT3 FNBP4 FOXO1 FOXO3 FOXO4 FOXP1 FRK FYN FZD5 GALM

GATA3 GNA11 GNA13 GNAQ GNAS GPC6 GPR157 GRK6 GSK3B HCK

HDAC1 HDAC4 HDAC7 HIPK4 HIST1H1C HIST1H1D HIST1H1E HIST1H2AC HIST1H2AG HIST1H2AL

HIST1H2AM HIST1H2BC HIST1H2BJ HIST1H2BK HIST1H2BO HIST1H3B HNF1A HRAS HSP90AA1 HSPA6

HSPD1 HTR1B ID2 ID3 IDH1 IDH2 IFI6 IGF1R IGSF21 IKBKB

IKBKE IKBKG IL3RA IL6R IL7 IL7R ILF2 IRAK1 IRAK4 IRF1

IRF4 IRF8 ITK JAK1 JAK2 JAK3 JUN KALRN KCNH2 KCNH5

KDR KIF4B KIFAP3 KIT KLF6 KLHL6 KMT2A KMT2B KMT2C KRAS

LCK LEF1 LILRB1 LRRC34 LYN MAF MALT1 MAP2K1 MAP2K2 MAP2K4

MAP3K1 MAP3K13 MAP3K14 MAP3K6 MAP3K7 MAPK1 MAPK3 MCL1 MDH1B MDM2

MDM4 MED12 MEF2B MEF2C MET MGAT3 MIOS MISP MLH1 MPL

MTOR MUC1 MUC2 MYC MYCL MYCN MYD88 MYO6 NCAPG NF1

NF2 NFAT5 NFATC1 NFATC2 NFATC3 NFATC4 NFKBIA NHSL2 NKG7 NKX2-2

NMT1 NOTCH1 NOTCH2 NPM1 NR0B1 NRAS NTRK1 NTRK2 NTRK3 P2RY8

PAX5 PCDH20 PCLO PDCD1 PDCD11 PDCD1LG2 PDGFRA PDGFRB PEX2 PIK3CA

PIK3CD PIK3CG PIK3R1 PIK3R2 PIM1 PLCB1 PLCG1 PLCG2 POU2AF1 POU2F2

PPM1H PPP3CA PPP3CB PPP3CC PPP3R1 PPP3R2 PRDM1 PRKCB PRKCDBP PRKD2

PRND PTCH1 PTCH2 PTEN PTK2 PTPN1 PTPN11 PTPRC PTPRD AB11FIP5

RAF1 RARA RASSF1 RASSF5 RB1 RBP1 REL RET RFX7 RHOA

RHOT2 RICTOR RNASEH2B ROS1 RUNX1 SCPEP1 SDK1 SETBP1 SETD2 SF3B1

SGK1 SH3BP4 SLAMF7 SLC1A4 SMAD2 SMAD4 SMARCA1 SMARCA4 SMARCAL1 SMARCB1

SMARCD1 SMO SOCS1 SOCS2 SOCS3 SORCS3 SOS2 SOX10 SOX2 SOX3

PATA31A3 SRC STAB2 STAT1 STAT2 STAT3 STAT4 STAT5A STAT5B STAT6

STK11 SVOPL SYK TBL1X TBL1XR1 TCF3 TCL1A TERT TET1 TET2

TET3 TJP1 TLL2 TMCC1 TMPRSS2 TNFAIP3 NFRSF11A TNFRSF14 TNFRSF17 TNFRSF8

TOP1 TP53 TP63 TRAF2 TRAF3 TRAF5 TRAPPC10 TRRAP TSC1 TSC2

TSGA10 TTC28 TYK2 UBXN11 USO1 VAV1 VHL WDFY3 WIF1 WIPF2

WT1 XBP1 XPO1 YES1 ZNF813

===========================================================================================
